# Trends in the Ophthalmic Workforce and Eye Care Infrastructure in South India: Cross-Sectional Questionnaire Study

**DOI:** 10.2196/50921

**Published:** 2024-01-09

**Authors:** Srinivasa R Pallerla, Madhurima R Pallerla, Sannapaneni Krishnaiah

**Affiliations:** 1 Andhra Pradesh Right to Sight Society Hyderabad India; 2 Sri Jyothi Eye Clinic Hyderabad India; 3 LV Prasad Eye Institute Hyderabad India

**Keywords:** trends, human resources, infrastructure, eye care, South India

## Abstract

**Background:**

This study is part of broad-based research to determine the impact of blindness control activities in general and with special reference to the Andhra Pradesh Right to Sight Society (APRTSS) activities in the southern Indian states of Andhra Pradesh and Telangana. As part of the global “VISION 2020: The Right to Sight” initiative, the APRTSS was established in the undivided state of Andhra Pradesh in 2002. Since then, the APRTSS has been actively implementing the strategies of VISION 2020 to reduce visual impairment and blindness in the state.

**Objective:**

The availability and distribution of the eye care workforce are essential to reach the goals of VISION 2020: The Right to Sight, the global initiative to eliminate avoidable blindness. This study assessed the trends in the availability and distribution of eye health professionals and eye care infrastructure in 2 southern Indian states: Andhra Pradesh and Telangana.

**Methods:**

This cross-sectional study used a pretested questionnaire to gather data for the year from 2012 to 2013. Data for 2002 to 2003 were collected from available historical records. The questionnaires were pretested in a pilot study conducted before the main survey. Pretested questionnaires were administered to all eye care professionals—ophthalmologists (n=1712) and midlevel ophthalmic personnel (MLOP; n=1250)—eye care facilities with ≥10 inpatient beds or performing ≥100 cataract surgeries per annum (n=640), local nongovernmental eye care organizations (n=182), and international eye care organizations (n=10). Data were collected for 2 different time periods: the baseline year of 2002 to 2003 and the target year of 2012 to 2013. Data analysis was conducted using SPSS version 19.0.

**Results:**

The response rates were 81.1% (519/640) for eye care facilities, 96.1% (1645/1712) for ophthalmologists, and 67.6% (845/1250) for MLOP. From 2002-2003 to 2012-2013, there has been an increase in eye care facilities, from 234 to 519 (121.8%); ophthalmologists, from 935 to 1712 (83.1%); and MLOP, from 767 to 1250 (63%). The ophthalmologist:population ratio improved from 1:88,260 in 2002-2003 to 1:51,468 in 2012-2013. The MLOP:population ratio improved from 1:168,283 in 2002-2003 to 1:138,117 in 2012-2013 but still falls short of the ideal number.

**Conclusions:**

Both southern Indian states are able to meet the requirements for ophthalmologists and eyecare infrastructure as per the goals of VISION 2020. However, the number of MLOP falls short of the ideal ratio for the population. This study has some limitations. For example, most of the data collected through questionnaires were based on self-report, which might introduce bias due to memory recall or over or under-reporting of certain information. However, this was addressed by cross-checking the collected data with information from supplementary sources.

## Introduction

Blindness and visual impairment represent a major public health problem in India [1-4]. The major causes of blindness and visual impairment in Andhra Pradesh and Telangana include cataract, refractive errors, retinal diseases, glaucoma, and corneal opacities, as reported in the Andhra Pradesh Eye Diseases study [5]. To tackle the problem of blindness and visual impairment, we need adequate human resources and sufficient infrastructure in eye care. Since the global “VISION 2020: the Right to Sight” initiative was launched in 1999, there has been a lot of progress in not only lessening the burden of blindness and visual impairment but also increasing the number of skilled eye care professionals and eye care infrastructure [6,7].

In line with the global Vision 2020 initiative, the undivided Andhra Pradesh state (the state was divided into Andhra Pradesh and Telangana states in 2014) established the Andhra Pradesh Right to Sight Society (APRTSS) in 2002 to work toward the VISION 2020 goals. Since its formation, the APRTSS has coordinated closely with major stakeholders in eye care such as those in the government, nongovernmental organization (NGO), and private sectors. Its activities include human resource development, infrastructural strengthening, disease control, and advocacy. To determine the impact of APRTSS VISION 2020 activities, we carried out a research project collecting information about the APRTSS activities from the baseline year of 2002 to 2003—the year in which the APRTSS was established—and the target year of 2012 to 2013—after a period of 10 years.

As part of the aforementioned research project, we carried out a survey about the ophthalmic workforce and infrastructure to identify the trends over a period of 10 years. An evidence base is essential to understand trends in human resources for health [8]. However, no regular mechanism exists in India to collect data on human resource trends in the provision of eye care services [9]. This study fills that gap by identifying trends in eye care. The results of the survey will be helpful to identify gaps, strengthen the eye care facilities, and overcome the maldistribution of human resources and infrastructure, in order to achieve the goals of VISION 2020. This study assessed trends in the availability and distribution of eye health professionals and eye care infrastructure in 2 southern Indian states: Andhra Pradesh and Telangana.

## Methods

### Study Design

This cross-sectional study used a pretested questionnaire for the year 2012 to 2013. The data for the 2002-2003 period were collected from available historical records.

We used questionnaires in both electronic and hard copy formats to collect the data. The questionnaires were developed based on the 6 building blocks of the universal health care system [10].

### Ethical Considerations

This study was conducted as part of the research project on the “Impact of implementation of blindness control activities in the state of Andhra Pradesh,” which was approved by the ethics committee of the LV Prasad Eye Institute (reference number: LEC 09-13-097) and conducted in accordance with the tenets of the Declaration of Helsinki.

### Definitions

For the purpose of this study, an eye care facility was defined as any health care facility where ophthalmologist services are available. The eye care facilities were identified as secondary or tertiary eye care facilities. For the purpose of this study, secondary eye care was defined as any eye care facility having an ophthalmologist conducting cataract and basic minor surgical procedures. Tertiary eye care was defined as any eye care facility with secondary eye care services as well as at least one subspecialty such as cornea, glaucoma, retina, or oculoplasty. Eye care facilities were categorized as government eye care facilities if they were established and funded by the government or other public sources such as universities and public sector organizations. NGO eye care facilities functioned on a no-profit, no-loss basis. Eye care facilities with a profit motive, irrespective of whether owned by an individual or a group of people or agencies, were categorized as private eye care facilities.

### Inclusion Criteria

All eye care facilities with ≥10 inpatient beds or performing ≥100 cataract surgeries per annum were eligible.

### Questionnaire

The questionnaire had 4 sections. Each section was distributed to concerned eye care professionals both in electronic form and hard copy to obtain the data.

#### Section 1: Questionnaire for Eye Care Facilities

The questionnaire for eye care facilities (Multimedia Appendix 1) was distributed to the director, superintendent, administrator, or manager in charge of the care facility. It was completed to obtain information for both the baseline and target years. It contained questions ranging from the services available, human resources, infrastructure, training facilities for eye care professionals, and any other relevant data.

#### Section 2: Questionnaire for Ophthalmologists

The questionnaire for ophthalmologists (Multimedia Appendix 2) was sent to all ophthalmologists working in government, NGO, and private eye care facilities. It was intended to be completed both by email and in hard copies by surface mail. It contained questions about demographic details; whether the ophthalmologist performs surgeries; whether the ophthalmologist practices in any subspecialties such as anterior segment surgeries, glaucoma, or retina; the average number of cataract surgeries per month; the principal method followed during cataract surgeries; professional experience; academic activity; and any training undergone.

#### Section 3: Questionnaire for MLOP

The questionnaire for MLOP (Multimedia Appendix 3) was distributed to all optometrists, ophthalmic assistants, and nurses working in all government, NGO, and private eye care facilities. It contained questions to elicit information on knowledge, skills, experience, and special training undergone such as in contact lens practice, refresher training in retinoscopy methods, and biomedical training for equipment maintenance. We also collected information on how many refractions were conducted per month, how many pairs of spectacles were prescribed per month, any administrative work, and any research activities.

#### Section 4: Questionnaire for District Blindness Control Societies and NGOs in Eye Care

The questionnaire for district blindness control societies (DBCSs) and NGOs in eye care (Multimedia Appendix 4) was distributed to program managers to obtain information on the impact of the implementation of blindness control activities in the district. It contained 3 subsections: section A for program managers of DBCS, section B for NGOs in eye care, and section C for international NGOs in eye care who were active in the state.

#### Follow-Up

Follow-up mechanisms were instituted every 2 weeks after mailing the questionnaire to the various stakeholders, and reminders were sent at the 3rd month and again at the 6th month.

### Additional Data Sources

In addition to the data collected through questionnaires, we gathered information from the following sources: (1) member directory for the All India Ophthalmological Society and its website, (2) directory of the Andhra Pradesh Ophthalmological Society and its website, (3) directory of the Telangana Ophthalmological Society and its website, (4) directory of the Andhra Pradesh Paramedical Board, and (5) websites of leading eye care institutions.

The information obtained from these sources helped us cross-check the data received through the questionnaires from eye care facilities, ophthalmologists, MLOP, and DBCSs. The data collected were entered in Excel sheets by 2 different data operators and cross-checked for any typographical errors. The data were analyzed using SPSS version 19.0 (IBM Corp) for Windows.

## Results

### Participants

As per the inclusion criteria, a total of 640 eye care facilities were identified, and a questionnaire was sent to the directors or those in charge of the facilities. Of the 640 facilities, responses were received from 519. Table 1 shows the number of questionnaires distributed to the various participants and the response rates. All the DBCSs responded to the questionnaire, whereas the lowest response rate was from MLOP.

**Table 1:** Response rates for eye care facilities, eye care professionals, and eye care organizations.

Questionnaire recipient	Questionnaires distributed, n	Response rate, n (%)
Eye care facilities	640	519 (81.1)
Ophthalmologists	1712	1645 (96.1)
Midlevel ophthalmic personnel	1250	845 (67.6)
Local NGOs^a^	182	165 (90.7)
International NGOs	10	9 (90)
DBCSs^b^	23	23 (100)

^a^NGOs: nongovernmental organizations.

^b^DBCSs: district blindness control societies.

### Eye Care Facilities and Service Delivery

The number of eye care facilities in the undivided state increased from 234 in 2002-2003 to 519 in 2012-2013 (121.8% increase). From 2002-2003 to 2012-2013, there was a marginal increase in the number of eye care facilities in the government sector (44 to 58, 31.8%), there was a substantial increase in the NGO sector (105 to 165, 57.1%), and the highest increase was seen in the private sector (85 to 296, 248.2%; Table 2).

**Table 2:** Number of eye care facilities in the combined state of Andhra Pradesh in 2002-2003 and 2012-2013.

Type of facility	Facilities in 2002-2003, n	Facilities in 2012-2013, n
Government	44	58
NGO^a^	105	165
Private	85	296

^a^NGO: nongovernmental organization.

The number of eye care facilities delivering secondary eye care in the undivided state increased from 198 in 2002-2003 to 440 in 2012-2013 (122.2% increase), and the number of eye care facilities delivering tertiary care increased from 36 in 2002-2003 to 79 in 2012-2013 (119.4% increase). The secondary and tertiary eye care facilities experienced a large jump in number from 2002-2003 to 2012-2013, whereas there was no increase in the number of tertiary eye care facilities in the government sector for the same period (Table 3).

Of 519 eye care facilities, 455 facilities (87.7%) were offering patient care services exclusively. Only 17% (88/519) of eye care facilities offered training facilities for eye care professionals and eye bank services in addition to patient care.

Regarding the eye care workforce, there was a substantial increase in the number of ophthalmologists in both southern Indian states. There was an insufficient increase in MLOP to meet the need. There was a large jump in the number of eye care managers, mostly in NGO and private eye care facilities (Table 4).

**Table 3:** Increase in secondary and tertiary eye care facilities from 2002 to 2012 by sector.

Eye care facility sector		Facilities in 2002-2003, n	Facilities in 2012-2013, n	Increase, %	*P* value
**Secondary**					<.001
	Government	34	48	41	
	NGO^a^	88	139	58	
	Private	76	253	233	
	All secondary	198	440	122	
**Tertiary**					.009
	Government	10	10	0	
	NGO	17	26	53	
	Private	9	43	378	
	All tertiary	36	79	119	

^a^NGO: nongovernmental organization.

**Table 4:** Eye care workforce in the 2002-2012 period.

Job role		Andhra Pradesh, n		Telangana, n		Both states, n		Increase, %
		2002	2012	2002	2012	2002	2012	
**Ophthalmologists**								
	Professor or senior consultant^a^	132	288	146	338	278	626	125
	Assistant professor or junior consultant^b^	257	364	248	467	505	831	64
	Ophthalmologists acting as superintendents or directors	69	148	83	107	152	255	67.8
	All ophthalmologists	458	800	477	912	935	1712	83.1
**Midlevel** **ophthalmic personnel (MLOP)**								
	Optometrists, refractionists, ophthalmic assistants, vision technicians	272	410	238	472	510	882	72.9
	Ophthalmic nurses and general nurses working in eye care facilities	58	111	72	130	257	368	43.2
	All MLOP	330	521	310	602	767	1250	63
Eye care managers		69	163	83	244	152	407	167.8

^a^Ophthalmologists with ≥10 years of experience.

^b^Ophthalmologists with ≤10 years of experience.

The ophthalmologist:population ratio ranged from 1:6309 in Hyderabad district, which is the capital area, to 1:193,822 in Nalgonda district (Table 5). This shows there was a maldistribution of ophthalmologists among the districts in the state. The ratio of optometrists and allied personnel to the population ranged from 1:66,209 in Ranga Reddy district to 1:221,173 in Guntur district. Overall, the ophthalmologist:population ratio in the state was 1:49,404, which appears to be optimal as per the VISION 2020 guidelines.

We looked at the number of eye care beds available for the population, and this improved from an average of 1:17,457 in 2002-2003 to an average of 1:13,877 in 2012-2013 (Table 6). There was also a lot of variation in the availability of eye care beds among the districts; for example, in Hyderabad district, 1 eye care bed was available for 3805 persons, compared with 1 eye care bed for 30,014 persons in Karimnagar. The total number of eye care beds increased from 4339 in 2002-2003 to 6103 in 2012-2013 (40.6% increase). On average, 1 ophthalmologist was available per 100,000 people/6 eye care beds in 2002-2003, which increased to an average of 2 ophthalmologists per 100,000 people/7 eye care beds in 2012-2013. A greater number of ophthalmologists per 100,000 population will improve the accessibility and availability of ophthalmologists to the public.

**Table 5:** Human resources in eye care in the districts of undivided Andhra Pradesh.

District name	Population, n		Ophthalmologists, n		Ophthalmologist:population ratio		MLOP^a^, n		MLOP:population ratio	
	2002-2003^b^	2012-2013^c^	2002-2003	2012-2013	2002-2003	2012-2013	2002-2003	2012-2013	2002-2003	2012-2013
Adilabad	2,479,347	2,741,239	N/A^d^	22	N/A	1:124,601	N/A	24	N/A	1:114,218
Hyderabad	3,686,460	3,943,323	N/A	625	N/A	1:6309	N/A	12	N/A	1:328,610
Karim Nagar	3,477,079	3,776,269	N/A	42	N/A	1:89,911	N/A	31	N/A	1:121,815
Khammam	2,565,412	2,797,370	N/A	30	N/A	1:93,245	N/A	10	N/A	1:279,737
Mahbub Nagar	3,506,876	4,053,028	N/A	18	N/A	1:225,168	N/A	28	N/A	1:144,751
Medak	2,662,296	3,033,288	N/A	14	N/A	1:216,663	N/A	20	N/A	1:151,664
Nalgonda	3,238,449	3,488,809	N/A	18	N/A	1:193,822	N/A	27	N/A	1:129,215
Nizamabad	2,342,803	2,551,335	N/A	29	N/A	1:87,977	N/A	19	N/A	1:134,280
Ranga Reddy	3,506,670	5,296,741	N/A	99	N/A	1:53,502	N/A	80	N/A	1:66,209
Warangal	3,231,174	3,512,576	N/A	55	N/A	1:63,865	N/A	39	N/A	1:90,066
Anantapur	3,639,304	4,081,148	N/A	45	N/A	1:90,692	N/A	24	N/A	1:170,047
Chittoor	3,735,202	4,174,064	N/A	44	N/A	1:94,865	N/A	23	N/A	1:181,481
East Godavari	4,872,622	5,154,296	N/A	93	N/A	1:55,422	N/A	37	N/A	1:139,305
Guntur	4,405,521	4,887,813	N/A	41	N/A	1:119,214	N/A	22	N/A	1:222,173
Kadapa	2,573,481	2,882,469	N/A	23	N/A	1:125,324	N/A	20	N/A	1:144,123
Krishna	4,218,416	4,517,398	N/A	108	N/A	1:41,827	N/A	22	N/A	1:205,336
Kurnool	3,512,266	4,053,463	N/A	57	N/A	1:71,113	N/A	30	N/A	1:135,115
Nellore	2,659,661	2,963,557	N/A	58	N/A	1:51,095	N/A	22	N/A	1:134,707
Prakasam	3,054,941	3,397,448	N/A	47	N/A	1:72,286	N/A	34	N/A	1:99,924
Srikakulam	2,528,491	2,703,114	N/A	11	N/A	1:245,737	N/A	20	N/A	1:135,155
Visakhapatnam	3,789,823	4,290,589	N/A	171	N/A	1:25,091	N/A	23	N/A	1:186,547
Vizianagaram	3,789,823	2,344,474	N/A	15	N/A	1:156,298	N/A	23	N/A	1:101,933
West Godavari	3,796,144	3,936,966	N/A	47	N/A	1:83,765	N/A	23	N/A	1:171,172
All districts	7,572,7541	8,458,0777	858^e^	1712	1:88,260	1:49,404	450^e^	613	1:123,535	1:137,978

^a^MLOP: midlevel ophthalmic personnel.

^b^Census 2001 [11].

^c^Census 2011 [12].

^d^N/A: not available.

^e^Approximate number from supplementary records.

**Table 6:** Population and number of eye care beds by district.

District name	Population, n		Eye care beds, n		Eye care bed:population ratio	
	2002-2003	2012-2013	2002-2003	2012-2013	2002-2003	2012-2013
Adilabad	2,479,347	2,737,738	207	265	1:11,978	1:10,331
Hyderabad	3,686,460	4,010,238	855	1054	1:4312	1:3805
Karimnagar	3,477,079	3,811,738	102	127	1:34,089	1:30,014
Khammam	2,565,412	2,798,214	97	187	1:26,448	1:14,964
Mahbub Nagar	3,506,876	4,042,191	144	184	1:24,353	1:21,968
Medak	2,662,296	3,031,877	87	87	1:30,601	1:34,849
Nalgonda	3,238,449	3,483,648	198	228	1:16,356	1:15,279
Nizamabad	2,342,803	2,552,073	142	197	1:16,499	1:12,955
Ranga Reddy	3,506,670	5,296,396	127	242	1:27,612	1:21,886
Warangal	3,231,174	3,934,842	242	367	1:13,352	1:10,722
Anantapur	3,639,304	4,083,315	182	262	1:19,996	1:15,585
Chittoor	3,735,202	4,170,468	123	144	1:30,367	1:28,962
East Godavari	4,872,622	5,151,549	192	372	1:25,378	1:13,848
Guntur	4,405,521	4,889,320	207	277	1:21,283	1:17,651
Kadapa	2,573,481	2,884,524	152	297	1:16,931	1:9712
Krishna	4,218,416	4,529,009	102	170	1:41,357	1:26,641
Kurnool	3,512,266	4,046,601	107	162	1:32,825	1:24,979
Nellore	2,659,661	2,966,082	93	112	1:28,599	1:26,483
Prakasam	3,054,941	3,392,764	220	223	1:13,886	1:15,214
Srikakulam	2,528,491	2,699,471	147	267	1:17,201	1:10,110
Visakhapatnam	3,789,823	4,288,113	205	330	1:18,487	1:12,994
Vizianagaram	3,789,823	2,342,868	132	217	1:28,711	1:10,797
West Godavari	3,796,144	3,934,782	276	331	1:13,754	1:11,888
All districts	75,727,541	84,665,533	4339	6103	1:17,457	1:13,877

## Discussion

### Principal Findings

Estimates indicate there are 4.95 million people who are blind (0.36% of the total population), 35 million people who are visually impaired (2.55%), and 0.24 million children who are blind in India [13]. Cataract and refractive errors remain the major causes of blindness and visual impairment, respectively, in India [13-16]. Cataract is responsible for nearly two-thirds of the blindness load in the older population in India [1-4], and one-fifth of blindness is due to uncorrected refractive errors [1-3]. There have been significant improvements in the field of blindness prevention, management, and control since the “VISION 2020: The Right to Sight” initiative [17]. In view of this background, India needs a pool of well-qualified, skilled, and optimal eye care professionals and sufficient infrastructure to eliminate avoidable and needless blindness and visual impairment.

The global advisory committee for VISION 2020 recommended a set of criteria for human resources and infrastructure based on expert consensus of the number of cataract procedures that could be performed by a surgeon per year under optimal conditions and the number of beds required for the same per 1 million population [9]. It was assumed that at least 50 procedures per bed per year could be optimally performed. Based on these assumptions, the following norms were recommended: 1 ophthalmologist per 50,000 population, 1 MLOP per 50,000 population, and 1 eye care bed per 20,000 population.

In this study, the ophthalmologist:population ratio in 2002-2003 was 1:88,822, and in 2012-2013, it reached 1:51,416. The state had almost reached the optimal ophthalmologist:population ratio. Previous data show that the national average ophthalmologist:population ratio is 1:107,000, ranging from 1:9000 in some regions to 1:608,000 in some areas [9]. There was a decrease in the percentage of ophthalmologists in the government sector and virtually no change in the percentage of ophthalmologists in the NGO sector. In addition, there was a substantial increase in the number of ophthalmologists in the private sector from 2002-2003 to 2012-2013. Some of the ophthalmologists, who were mainly working in the private sector, offered their services for a few hours a day or 1 to 2 days a week to NGO eye care facilities, either free or for a fee. As per our study definition, these ophthalmologists who were providing their services part-time for the NGO eye care facilities were treated as working in the private sector only. Hence, the number of ophthalmologists working in the NGO sector appears to be under-reported when compared with that of other sectors.

As per VISION 2020, there should be 20 ophthalmologists and 50 beds per 1 million population [18]. The importance of the ophthalmologist:population ratio is that it can serve as a guide to forecast ophthalmic manpower requirements [19]. As per the norm, the number of available eye care beds is sufficient, and there is no need to increase the number of eye care beds; in addition, there is a shift toward day surgeries for cataract [8].

The distribution of ophthalmologists was skewed toward urban areas. Due to the lack of educational facilities for their children and other lifestyle-related infrastructure in underdeveloped areas, ophthalmologists and private eye care facilities tend to be established in developed urban areas. In the Telangana region, the majority of the ophthalmologists were practicing in Hyderabad City, whereas in coastal Andhra, many of the ophthalmologists were practicing in the urban areas of Visakhapatnam and Vijayawada. Compared with the coastal Andhra region, this phenomenon of ophthalmologists working in urban areas was more pronounced in the Telangana region. As urban areas became more crowded with ophthalmologists, there was a trend that some ophthalmologists started their practices in smaller towns in 2012-2013. In 2002-2003, ophthalmologists were mainly present in the district headquarters and major population areas. This trend changed in 2012-2013 when more eye care facilities were opened in less populated areas.

Murthy et al [20] reported that 69% of ophthalmologists worked in the private and NGO sectors, while 31% were working in the government sector. In this study, 88% of ophthalmologists were working in the private and NGO sectors, and the remaining 12% were working in the government sector. In this study, the majority of the ophthalmologists in the government sector were working in teaching institutions rather than in district and subdistrict hospitals similar to that reported by Murthy et al [20]. In this study, we found the average number of surgeries performed by surgeons in the NGO sector was significantly higher than that in other sectors in both the baseline and target years. After the ophthalmologists in the NGO sector, ophthalmologists in the government sector were performing more surgeries than those in the private sector.

Ophthalmologists with less than 10 years of experience were performing more cataract surgeries than those with more than 10 years of experience (*P*=.001). This may be because some of the senior ophthalmologists were involved in teaching and research. This finding corroborates the fact that nonteaching ophthalmologists were performing more cataract surgeries than their teaching counterparts.

The state should ideally have 1693 MLOP for its population of 84.6 million. The state needs 1080 more MLOP to reach this number. The majority of the MLOP either were not trained in streak retinoscopy or did not have access to streak retinoscopes. There is a need for a strategy to ensure that all MLOP can perform streak retinoscopy.

There were many reasons for the increase in the number of both secondary and tertiary eye care facilities in all 3 sectors—government, NGO, and private—from 2002-2003 to 2012-2013. The number of eye care facilities as well as the number of eye care professionals increased during this period. The highest increase in eye care facilities (248%) was seen in the private sector due to the establishment of many institutions for eye care professionals in both government and NGO sectors. People trained at these institutes either were absorbed into the private sector or started their own practice, because there was no recruitment in the government sector or minimal opportunities in the NGO sector. This is the reason why the number of secondary eye care facilities increased more than tertiary eye care facilities. Another reason was, compared with other fields in medical practice, it is easier to start a solo practice in eye care, as it does not depend on cooperation from other medical streams. For example, to start a general surgery or orthopedics practice, one requires the services of an anesthetist. To start a pediatric practice, good laboratory services are required. Of the 519 eye care facilities functioning in 2012-2013, 253 (48.7%) were from the private sector. This was similar to the findings reported by Murthy et al [1], in which more than one-half of the eye care facilities belonged to the private sector.

### Limitations

This study has some limitations. Most of the data collected through questionnaires were based on self-report, which might introduce bias due to memory recall or over or under-reporting of certain information. However, this was addressed by cross-checking the collected data with information from the supplementary sources mentioned in the Methods section.

### Conclusion

Regarding human resources, there was a substantial increase in the number of ophthalmologists, particularly in the private sector. In fact, the percentage of ophthalmologists in the government sector decreased from the baseline year to the target year, whereas in the NGO sector, it remained the same.

Though all 3 sectors—government, NGO, and private—showed an increase in the number of eye care facilities from the baseline year to the target year, substantial increases were seen in the private sector and, to some extent, in the NGO sector. Most of the eye care facilities offered patient care services only. The outpatient services and inpatient services were also higher in 2012-2013 in all 3 sectors, but the NGO sector contributed a major share, followed by the private sector. Regarding outreach activities, the NGO sector dominated the services, to the extent of 80%-97%. One NGO facility collected the majority of eyes for corneal transplantation, and the remaining eye care facilities in the government, NGO, or private sector showed very little improvement in their collection of eyes.

Regarding eye care infrastructure, there was a 41% increase in the number of beds available for eye care, and this increase was mainly due to the NGO sector, followed by the private sector. The average number of surgeries per surgeon per annum was highest in the NGO sector, followed by the government sector. There was a major shortage of MLOP in the state to attain the ideal ratio of 1 MLOP per 50,000 population. To attain the ideal number of MLOP, there is an urgent need to increase the number of training facilities for MLOP. Overall, the functioning of the DBCSs for planning and supervising district eye care programs was satisfactory.

## Appendix

Multimedia Appendix 1 Questionnaire for eye care facilities.

Multimedia Appendix 2 Questionnaire for ophthalmologists.

Multimedia Appendix 3 Questionnaire for midlevel ophthalmic personnel.

Multimedia Appendix 4 Questionnaire for district blindness control societies (DBCSs) and nongovernmental organizations (NGOs) in eye care.

## Abbreviations

APRTSS: Andhra Pradesh Right to Sight Society
DBCS: district blindness control society
MLOP: midlevel ophthalmic personnel
NGO: nongovernmental organization

## References

[1] Murthy GV, Ellwein LB, Gupta S, Tanikachalam K, Ray M, Dada VK (2001). A population-based eye survey of older adults in a rural district of Rajasthan: II. Outcomes of cataract surgery. Ophthalmology.

[2] Thulasiraj RD, Rahamathulla R, Saraswati A, Selvaraj S, Ellwein LB (2002). The Sivaganga eye survey: I. Blindness and cataract surgery. Ophthalmic Epidemiol.

[3] Nirmalan PK, Thulasiraj RD, Maneksha V, Rahmathullah R, Ramakrishnan R, Padmavathi A, Munoz SR, Ellwein LB (2002). A population based eye survey of older adults in Tirunelveli district of south India: blindness, cataract surgery, and visual outcomes. Br J Ophthalmol.

[4] Thulasiraj RD, Nirmalan PK, Ramakrishnan R, Krishnadas R, Manimekalai TK, Baburajan NP, Katz J, Tielsch JM, Robin AL (2003). Blindness and vision impairment in a rural south Indian population: the Aravind Comprehensive Eye Survey. Ophthalmology.

[5] Dandona R, Dandona L (2001). Review of findings of the Andhra Pradesh Eye Disease Study: policy implications for eye-care services. Indian J Ophthalmol.

[6] Foster A, Resnikoff S (2005). The impact of Vision 2020 on global blindness. Eye (Lond).

[7] Kuper H, Foster A (2006). Impact of VISION 2020 on global blindness. Can J Ophthalmol.

[8] Diallo K, Zurn P, Gupta N, Dal Poz M (2003). Monitoring and evaluation of human resources for health: an international perspective. Hum Resour Health.

[9] Kumar R (1993). Ophthalmic manpower in India--need for a serious review. Int Ophthalmol.

[10] (2010). Monitoring the building blocks of health systems: a handbook of indicators and the measurement strategies. World Health Organization.

[11] Census 2001. Registrar General of India, Government of India.

[12] Andhra Pradesh Population Census 2011 | Andhra Pradesh Religion, Caste Data - Census 2011. Census India.

[13] (2020). National Programme for Control of Blindness and Visual Impairment.

[14] Wadhwani M, Vashist P, Singh SS, Gupta V, Gupta N, Saxena R (2020). Prevalence and causes of childhood blindness in India: A systematic review. Indian J Ophthalmol.

[15] Misra N, Khanna RC (2020). Commentary: Rapid assessment of avoidable blindness and diabetic retinopathy in India. Indian J Ophthalmol.

[16] Abdulhussein D, Abdul Hussein M (2023). WHO Vision 2020: Have we done it?. Ophthalmic Epidemiol.

[17] Resnikoff S, Pararajasegaram R (2001). Blindness prevention programmes: past, present, and future. Bull World Health Organ.

[18] (1997). Global initiative for the elimination of avoidable blindness WHO/PBL/97. World Health Organization.

[19] Trobe JD, Kilpatrick KE (1983). Ophthalmology manpower: shortfall or windfall?. Surv Ophthalmol.

[20] Murthy GVS, Gupta SK, Bachani D, Tewari HK, John N (2004). Human resources and infrastructure for eye care in India: current status. Natl Med J India.

